# Deciphering the molecular specificity of phenolic compounds as inhibitors or glycosyl acceptors of β-fructofuranosidase from *Xanthophyllomyces dendrorhous*

**DOI:** 10.1038/s41598-019-53948-y

**Published:** 2019-11-25

**Authors:** Mercedes Ramirez-Escudero, Noa Miguez, Maria Gimeno-Perez, Antonio O. Ballesteros, Maria Fernandez-Lobato, Francisco J. Plou, Julia Sanz-Aparicio

**Affiliations:** 1 Macromolecular Crystallography and Structural Biology Department, Institute of Physical-Chemistry Rocasolano, CSIC, Serrano 119, 28006 Madrid, Spain; 2 Institute of Catalysis and Petrochemistry, CSIC, Marie Curie 2, 28049 Madrid, Spain; 3 Centre of Molecular Biology Severo Ochoa, UAM-CSIC, Autonomous University of Madrid, 28049 Madrid, Spain

**Keywords:** Biochemistry, Biotechnology, Chemical biology, Computational biology and bioinformatics

## Abstract

Enzymatic glycosylation of polyphenols is a tool to improve their physicochemical properties and bioavailability. On the other hand, glycosidic enzymes can be inhibited by phenolic compounds. In this work, we studied the specificity of various phenolics (hydroquinone, hydroxytyrosol, epigallocatechin gallate, catechol and *p*-nitrophenol) as fructosyl acceptors or inhibitors of the β-fructofuranosidase from *Xanthophyllomyces dendrorhous* (pXd-INV). Only hydroquinone and hydroxytyrosol gave rise to the formation of glycosylated products. For the rest, an inhibitory effect on both the hydrolytic (H) and transglycosylation (T) activity of pXd-INV, as well as an increase in the H/T ratio, was observed. To disclose the binding mode of each compound and elucidate the molecular features determining its acceptor or inhibitor behaviour, ternary complexes of the inactive mutant pXd-INV-D80A with fructose and the different polyphenols were analyzed by X-ray crystallography. All the compounds bind by stacking against Trp105 and locate one of their phenolic hydroxyls making a polar linkage to the fructose O2 at 3.6–3.8 Å from the C2, which could enable the ulterior nucleophilic attack leading to transfructosylation. Binding of hydroquinone was further investigated by soaking in absence of fructose, showing a flexible site that likely allows productive motion of the intermediates. Therefore, the acceptor capacity of the different polyphenols seems mediated by their ability to make flexible polar links with the protein, this flexibility being essential for the transfructosylation reaction to proceed. Finally, the binding affinity of the phenolic compounds was explained based on the two sites previously reported for pXd-INV.

## Introduction

Plant polyphenols constitute a large group of substances whose regular consumption may help to delay the appearance of several degenerative pathologies, including Parkinson’s and Alzheimer’s diseases, cancer, chronic inflammatory disease or atherosclerosis^1,2^. In nature, polyphenols can be found conjugated to sugars^3^, which play an important role in their solubility^4,5^, stability^6,7^, bioavailability^8^ and bioactivity^9^. In fact, glycosylation is a tool to improve the bioavailability and pharmaceutical properties of polyphenols^10–14^. Compared with traditional chemical methods, biocatalytic processes ‒using glycosidases or glycosyltransferases‒ offer numerous advantages for polyphenol glycosylation^15–17^, including regio- and stereospecificity, mild reaction conditions and global sustainability^18^.

However, it is well reported that polyphenols may also inhibit glycosidic enzymes^19^. This process is critical in the production of lignocellulosic bioethanol, as the phenolic compounds released from lignin (generated after biomass pre-treatment) usually inhibit glycosidases thus lowering the production of fermentable sugars^20–22^. Glycosidase inhibition by polyphenols has also significant implications in health. The inhibition of the human amylolytic system, composed of α-amylases and α-glucosidases, could help to control the rate of glucose release after ingestion of starch-containing foods. In this context, Gong *et al*. described the inhibition of human α-glucosidase by hesperetin^23^. Simsek *et al*. demonstrated that several dietary polyphenols such as (-)-epigallocatechin gallate (EGCG) were able to inhibit the two α-glucosidases (maltase-glucoamylase and sucrose-isomaltase) located on the small intestinal brush border, which could lead to a slower digestion of starchy foods and to an improved glycemic response^24^. The above polyphenols could represent an alternative to current α-glucosidase inhibitors in the market for the treatment of type II diabetes. Apart from energy uptake, glycosidases are involved in other critical cellular processes in biological systems such as catabolism or post-translational glycosylation of proteins^25^, which reinforces the interest in all aspects related to the inhibition of these enzymes.

*Xanthophyllomyces dendrorhous* β-fructofuranosidase (Xd-INV, EC 3.2.1.26) is a highly glycosylated dimeric enzyme that belongs to CAZy family GH32 and hydrolyzes sucrose and various fructooligosaccharides (FOS) and fructans releasing fructose^26^. It also catalyzes the synthesis of short-chain FOS, in which the fructosyl moiety is transferred to the sucrose skeleton. Whereas the majority of the reported fructosylating enzymes form β(2 → 1) or β(2 → 6) linkages between fructosides, Xd-INV is able to transfer the fructosyl unit to the glucose moiety of sucrose, generating neo-FOS with a levan-type structure, along with minor amounts of inulin-type β(2 → 1)FOS^27,28^. Moreover, Xd-INV is also capable to fructosylate other carbohydrates containing glucose^29^ yielding novel hetero-fructooligosaccharides with potential application as functional foods or nutraceuticals.

The molecular basis of the broad specificity of Xd-INV activity was previously assessed by crystallography^30,31^. The analysis of its D80A inactivated variant complexed with a series of different oligosaccharides revealed that the enzyme presented at least four binding subsites at the catalytic pocket. Furthermore, two alternative binding modes were observed from subsite +2 explaining its versatility in binding different types of substrates. Thus, the aromatic side-chain of Trp105 makes a preferred and plastic hydrophobic platform that allocates neoFOS or β(2 → 6) related oligosaccharides, whilst the flexible Glu334-Asn343 loop makes a secondary binding site for β(2 → 1) inulin-type substrates, mostly through polar interactions. In a recent work, we found that the phenolic antioxidant hydroxytyrosol was able to profit from this bivalent binding mode, generating two fructosylated derivatives^32^. This feature was further exploited to modulate the enzyme regiospecificity by mutagenesis of particular residues. This issue prompted us to explore in this work the activity of Xd-INV to glycosylate other biologically relevant polyphenolic compounds.

It is worth noting that the inhibition of β-fructofuranosidases has been hardly investigated^33^, probably due to the inexistence of such enzymes in the animal kingdom, except for the silkworm *Bombyx mori*^34^ and the coleopteran *Sphenophorus levis*^35^. However, the crucial role of β-fructofuranosidases in microorganisms has been widely demonstrated^27,36–38^.

Thus, the goal of the present work was to assess the behaviour of a series of phenolic compounds as fructosyl acceptors or inhibitors of the β-fructofuranosidase from *X. dendrorhous*. We have evaluated the effect of such compounds on its hydrolysis to transfructosylation ratio. The specific functionality as acceptor or inhibitor for each compound was inspected by solving the crystal structure of the corresponding complexes, and disclosing the enzymatic mechanisms at the molecular level. Our final goal was to increase the knowledge for the design of efficient biocatalysts for the production of bioactive polyphenol glycosides, as well as decipher at the molecular level the main features governing the interaction of polyphenols with the active-site of β-fructofuranosidases. This information could be of great value for the study of structure-function relationships of other glycosidases.

## Results and Discussion

### Fructosylation of phenolic compounds with pXd-INV

**Figure 1:**
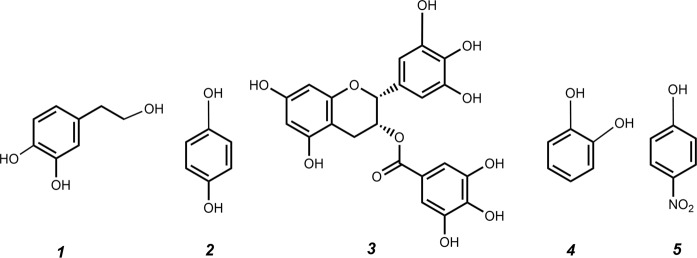
Structure of the phenolic compounds studied in this work. (1) Hydroxytyrosol (HT); (2) Hydroquinone (HQ); *(3)* (-)-Epigallocatechin gallate (EGCG); (4) Catechol (CAT); (5) *p*-Nitrophenol (PNP).

Besides catalyzing the hydrolysis of sucrose and the synthesis of neo-FOS^26^, the β-fructofuranosidase Xd-INV can also accomplish the fructosylation of several carbohydrates^29^ and other non-sugar acceptors such as hydroxytyrosol (HT)^32^. In order to expand the range of Xd-INV acceptors, a variety of phenolic compounds were tested and compared with HT: hydroquinone (HQ), (-)-epigallocatechin gallate (EGCG), catechol and *p*-nitrophenol (Fig. 1). Reactions were performed at 60 °C with 100 g/L of sucrose as fructosyl donor, 20 g/L of the putative acceptor and 0.72 U/mL of the β-fructofuranosidase, heterologously expressed in *Pichia pastoris* (pXd-INV)^39^. Control reactions in absence of acceptor or sucrose were carried out under the same conditions. Reaction mixtures were analyzed by TLC and HPLC.

**Figure 2:**
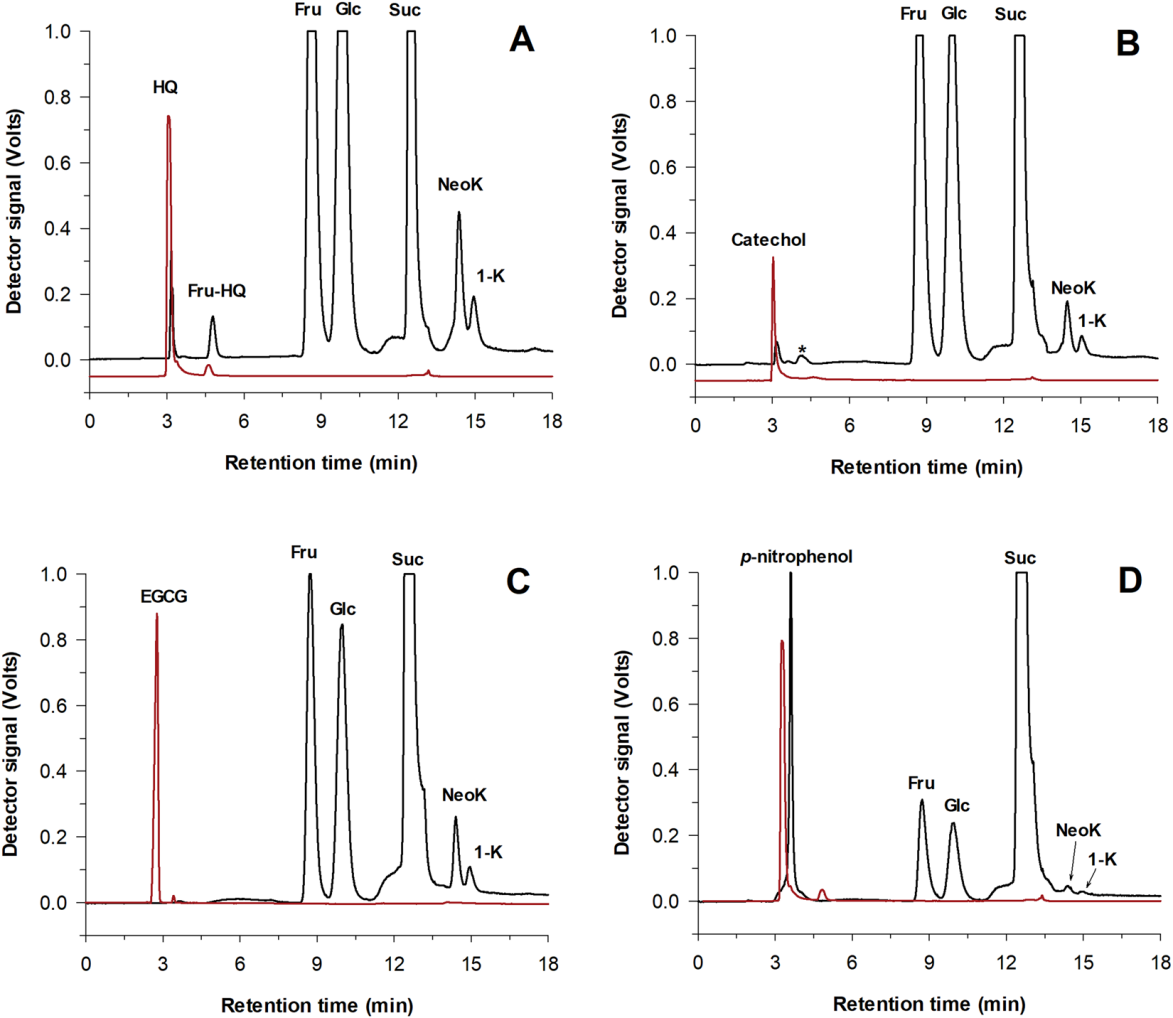
Effect of different phenolic compounds on pXd-INV activity. HPLC analysis of the reactions in presence of: (**A**) hydroquinone (HQ); (**B**) catechol (CAT); (**C**) epigallocatechin gallate (EGCG); (**D**) *p*-nitrophenol (PNP). The ELSD (black line) and UV (red line, 296 nm for **A**,**D**; 241 nm for **B**,**C**) detector signals are represented. Reaction conditions: 100 g/L of sucrose, 20 g/L of phenolic compound, 100 mM sodium acetate buffer (pH 5.0), 60 °C. Peaks: (Fru-HQ) Fructosyl-hydroquinone; (Fru) Fructose; (Glc) Glucose; (Suc) Sucrose; (NeoK) Neokestose; (1-K) 1-Kestose; (*) Possible transfructosylation product of CAT.

In the preliminary analysis by TLC, we observed that, apart from HT^32^, the only compound that gave rise to the formation of a new glycosylation product was hydroquinone. This result was further confirmed by HPLC. Figure 2 illustrates the chromatograms (ELSD and UV signals) of the reactions in the presence of HQ, EGCG, catechol and *p*-nitrophenol, obtained after 2 h of reaction under the above experimental conditions. In the case of HQ, the appearance of a new peak with higher retention time than the acceptor indicated the fructosylation of this compound. For catechol, we detected a small peak in the ELSD chromatogram but it was not appreciable in the UV detector, indicating that the transfructosylation (if it occurs) was not significant. For EGCG and *p*-nitrophenol, no novel peaks were detected.

After purification of the HQ derivative by semipreparative HPLC, we confirmed HQ fructosylation by mass spectrometry (Supplementary Fig. S1). We observed a peak in the MS spectrum in positive mode at *m/z* 295.07 corresponding to the M + [Na]^+^ ion. Considering that the two phenolic OHs of hydroquinone are chemically equivalent, the synthesized compound must be 4-hydroxyphenyl-β-D-fructofuranoside. This compound was first obtained with the levansucrase from *Leuconostoc mesenteroides*^40^. Other types of HQ glycosylation have been achieved with several enzymatic systems^41,42^ and one of these glycosides (the β-glucoside arbutin) is being employed in the cosmeceutical industry as a skin lightening agent for the treatment of pigmentation disorders, as it inhibits the human tyrosinase activity^43–45^. The performance of the β-fructoside of HQ has been reported to be even superior to that of arbutin^40^.

**Figure 3:**
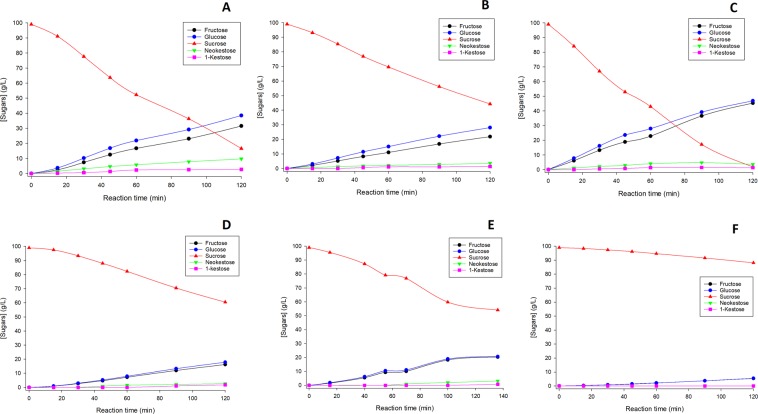
Progress of the hydrolytic and transfructosylation reactions in presence of the following phenolic compounds: (**A**) None (control reaction); (**B**) Hydroxytyrosol (HT); (**C**) Hydroquinone (HQ); (**D**) Epigallocatechin gallate (EGCG); (**E**) Catechol; (**F**) *p*-Nitrophenol. Reactions conditions: 100 g/L of sucrose, 20 g/L of phenolic compound,100 mM sodium acetate buffer (pH 5.0), 60 °C. The concentrations of fructose, glucose, sucrose, neokestose and 1-kestose are represented.

Figure 2 (ELSD detector, black lines) also illustrates that the presence of phenolic compounds alters both the hydrolytic and transfructosylation activities of pXd-INV. The sugars profile in presence of HQ was very similar to the one obtained in the control experiment in absence of phenolic derivatives (Fig. 3). In the case of catechol, a slight inhibitory effect on both the hydrolytic and transglycosylation activity was observed. This inhibition became significant when using EGCG and *p*-nitrophenol as acceptors (the peaks of monosaccharides and FOS were smaller than in the control experiments). These results indicated that phenolic compounds could produce different effects on the enzyme pXd-INV, acting as acceptors (HT, HQ) or inhibitors (catechol, EGCG and *p*-nitrophenol).

We studied in detail the effect of the assayed substances on the hydrolytic and transfructosylation (FOS formation) rates. We included HT in this analysis since we recently demonstrated that it is an efficient acceptor of pXd-INV^32^. The initial hydrolysis and transfructosylation rates were calculated as described in the Experimental Section. The data employed to calculate such rates is represented in Fig. 3. Table 1 summarizes the results obtained with the different phenolic compounds assayed.

**Table 1:** Effect of the assayed phenolic compounds on the hydrolytic and transfructosylation activity of pXd-INV.

Compound	Hydrolysis rate (mM min^−1^)	Transfructosylation rate (mM min^−1^)	H/T^a^
Control	1.45 ± 0.09	0.52 ± 0.06	2.76 ± 0.33
Hydroxytyrosol	1.02 ± 0.01	0.35 ± 0.01	2.92 ± 0.08
Hydroquinone	2.41 ± 0.05	0.55 ± 0.02	4.37 ± 0.15
EGCG	0.71 ± 0.03	0.07 ± 0.01	9.83 ± 0.78
Catechol	0.93 ± 0.05	0.06 ± 0.01	14.3 ± 1.3
*p*-Nitrophenol	0.23 ± 0.01	0.0035 ± 0.0003	65.3 ± 6.7

^a^Hydrolysis to transfructosylation ratio.

As shown in Table 1, transfructosylation rates decreased with all the investigated phenolic compounds, except for HQ in which the effect was almost negligible. The transfructosylation rate was 7.4-, 7.8- and 160-fold slower in presence of EGCG, catechol and *p*-nitrophenol, respectively, compared with the control experiment. The effect of the phenolics on the hydrolytic activity of pXd-INV was not so dramatic. It is worth noting that HQ even caused a slight increase in the hydrolytic activity of this enzyme, maintaining the transglycosylation activity. HQ could cause a local change in the active-site microenvironment, making it more hydrophilic. Combining both effects, the hydrolysis to transglycosylation ratio (H/T) of pXd-INV was highly dependent on the nature of the phenolic compound. Thus, the best acceptor of the substances assayed (HT) displayed a similar H/T ratio than the control experiment in absence of phenolics. However, the highest inhibitory substance (*p*-nitrophenol) increased the H/T 24-fold, indicating that these inhibitors must bind into the active site in a way that asymmetrically affects the hydrolytic and transfructosylation mechanisms of β-fructofuranosidases.

### Binding of the polyphenols to pXd-INV

**Figure 4:**
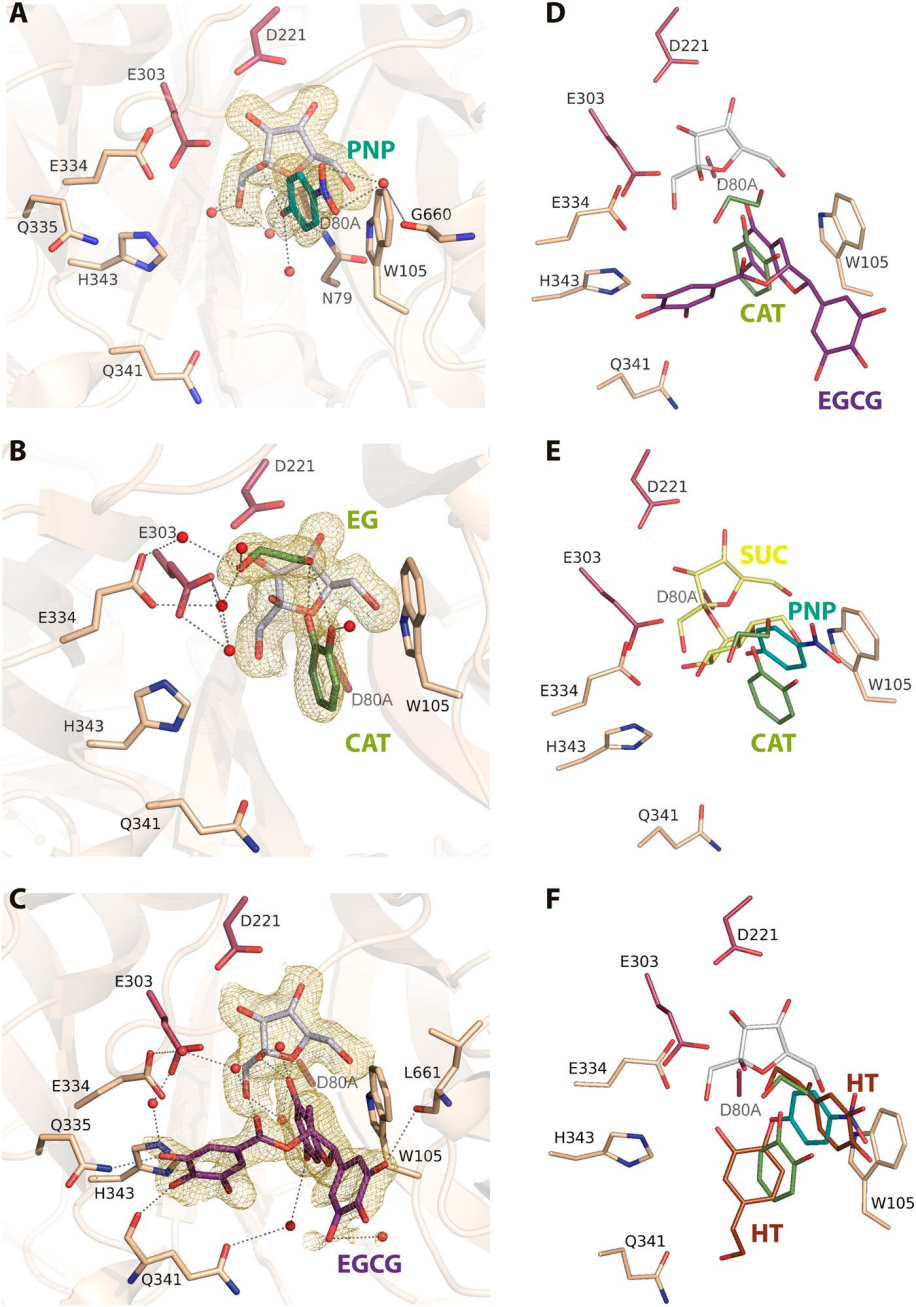
pXd-INV-D80A mutant complexes with phenolic inhibitors. A detail of the active site showing key residues for binding represented as sticks (the catalytic Asp221, Glu334 and the mutated D80A coloured in prune) and relevant water molecules as red spheres. The *2Fo-Fc* electron density at the bound molecules has been contoured at RMSD of 0.9–1 ơ. Crystals were soaked into β-D-fructose and then into: (**A**) *p*-nitrophenol (PNP), (**B**) catechol (CAT), (**C**) epigallocatechin gallate (EGCG). A trapped ethylene glycol (EG) molecule was found in the catechol-soaked crystals. Main polar interactions of each inhibitor with the residues at the active site are represented as dashed lines, those corresponding to fructose (white sticks) being common and omitted for clarity. (**D**) Structural superimposition of the inhibitors positions from the catechol (green) *vs*. the EGCG (purple) ‒soaked crystals, showing the common fructose at subsite ‒1 in white. The catechol is located at the same position that the diphenolic moiety of benzopyrane from EGCG. (**E**) Structural comparison of the bound catechol and *p*-nitrophenol positions, compared to the pXd-INV complexed with sucrose^30^, PDB code 5FIX. *p*-Nitrophenol is bound at a position close to glucose from sucrose, therefore competing with this donor substrate and blocking hydrolysis. (**F**) Structural comparison of the catechol and *p*-nitrophenol positions, compared to the two positions of the HT acceptor found in the reported pXd-INV-fructose complex^56^, PDB code 5NSL. Images created with software Pymol 1.7 (http://www.pymol.org/).

**Figure 5:**
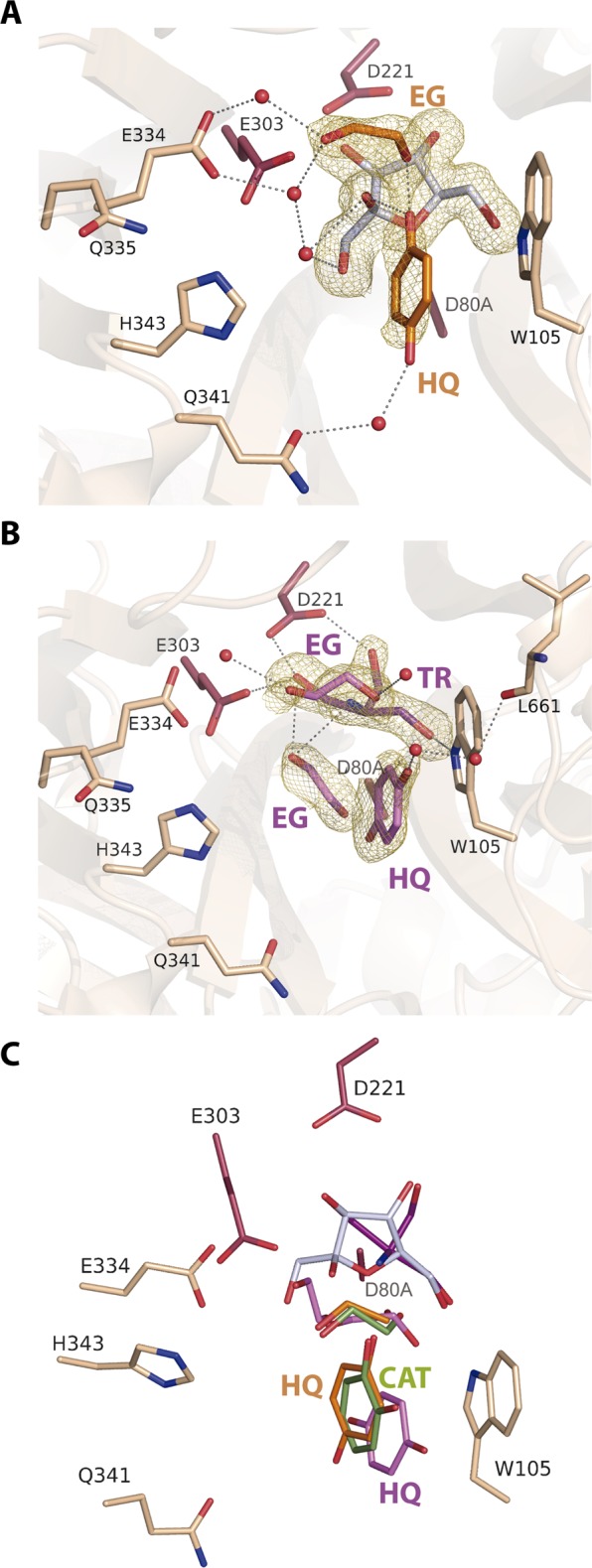
pXd-INV-D80A mutant complexes with hydroquinone as acceptor substrate. (**A**) Crystals soaked into fructose and HQ with a molecule of ethylene glycol trapped from the cryobuffer. (**B)** Crystals soaked into HQ, with two trapped ethylene glycol (EG) molecules and a molecule of Tris (TR) occupying the equivalent position of β-D-fructose in the ternary complexes. (**C**) Structural superimposition of the two complexes containing HQ (orange and violet) with the ternary complex with catechol (forest). Two positions are observed for HQ, one of them equivalent to catechol. Images created with software Pymol 1.7 (http://www.pymol.org/).

To disclose binding of the substrates to the active site, the pXd-INV nucleophile (aspartate) was mutated to alanine. Then, crystals from the inactivated pXd-INV-D80A enzyme were used for soaking experiments into β-D-fructose and the different polyphenols tested, to get the corresponding ternary intermediate complexes. The purpose was to analyze the binding mode of each compound that could elucidate the molecular basis directing to inhibition or, alternatively, their capability to act as acceptor substrates. Also, the crystals were soaked into hydroquinone in absence of fructose to investigate potential additional binding sites for this acceptor. The experimental data for all complexes are given in Table 2. All the experiments led to clear electron density that allowed unambiguous modelling of the bound molecules, as it is depicted in Figs. 4 and 5. In the ternary complexes with catechol and HQ, a molecule of ethylene glycol from the cryoprotectant solution was also found at the active site. In the HQ-soaked crystals, a tris(hydroxymethyl)aminomethane (Tris) molecule from the buffer was occupying the position of fructose found in the ternary complexes. On the other hand, the Asp to Ala mutation does not introduce any apparent change in the catalytic site with respect to the native enzyme.

**Table 2:** Crystallographic statistics. Values in parentheses are for the high-resolution shell.

Crystal data	pXd-INV-D80A/Fructose + *p*-Nitrophenol	pXd-INV-D80A/Fructose + Catechol	pXd-INV-D80A/Fructose + EGGC	pXd-INV-D80A/Fructose + Hydroquinone	pXd-INV-D80A/Hydroquinone
Space group	*P*2_1_2_1_2	*P*2_1_2_1_2	*P*2_1_2_1_2	*P2*_1_2_1_2	*P2*_1_2_1_2
**Unit cell parameters**					
a (Å)	74.51	74.72	74.85	74.56	74.58
b (Å)	205.65	204.74	205.97	205.95	205.62
c (Å)	146.51	147.27	145.17	146.16	145.37
**Data collection**					
Beamline	XALOC (ALBA)	XALOC (ALBA)	XALOC (ALBA)	XALOC (ALBA)	XALOC (ALBA)
Temperature (K)	100	100	100	100	100
Wavelength (Å)	0.9793	1.0419	0.9795	0.9795	0.9795
Resolution (Å)	84.17–1.73 (1.76–1.73)	119.60–1.80 (1.83–1.80)	118.17–2.03 (2.06–2.03)	48.72–1.85 (1.88–1.85)	83.94–1.85 (1.88–1.85)
**Data processing**					
Total reflections	1589757 (79091)	1406323 (66877)	982251 (48270)	1301543 (64491)	1296254 (64488)
Unique reflections	234346 (11421)	209132 (10286)	145475 (7129)	192124 (9418)	190741 (9345)
Multiplicity	6.8 (6.9)	6.7 (6.5)	6.8 (6.8)	6.8 (6.8)	6.8 (6.9)
Completeness (%)	100.0 (100.0)	100.0 (99.9)	100.0 (100.0)	100.0 (100.0)	100.0 (99.7)
Mean *I*/σ (*I*)	16.7 (3.1)	11.8 (2.7)	15.2 (3.5)	16.4 (3.3)	14.5 (2.9)
*R*_*merge*_^†^ (%)	6.2 (57.3)	8.6 (53.7)	8.0 (55.6)	6.9 (58.7)	7.9 (55.9)
*R*_*pim*_^††^ (%)	2.6 (23.3)	3.6 (22.6)	3.3 (23.0)	2.8 (24.1)	3.2 (22.7)
Molecules per ASU	2	2	2	2	2
**Refinement**					
R_work_/R_free_^†††^ (%)	16.3/17.8	16.4/17.2	16.8/18.8	16.0/18.0	15.4/17.5
**N° of atoms/average B (Å**^2^**)**					
Protein	9628/29.47	9610/23.50	9610/31.65	9610/26.95	9610/25.91
Carbohydrate and ligand molecules	987/52.76	841/44.95	1142/60.08	876/52.28	953/52.51
Water Molecules	1203/36.37	1299/35.08	955/40.61	971/34.93	1348/38.48
All atoms	11818/32.12	11750/26.31	11707/35.15	11457/29.56	11911/29.46
**Ramachandran plot (%)**					
Favoured	96.00	96.00	96.00	96.00	96.00
Outliers	0	0	0	0	0
**RMS deviations**					
Bonds (Å)	0.007	0.007	0.008	0.012	0.012
Angles (°)	1.443	1.330	1.534	1.776	1.755
PDB accession codes	6FJE	6S2H	6S2G	6S3Z	6S82

^†^R_merge_ = ∑_hkl_ ∑_i_ | I_i_(hkl) − [I(hkl)]|**/**∑_hkl_ ∑_i_ I_i_(hkl), where I_i_(hkl) is the ith measurement of reflection hkl and [I(hkl)] is the weighted mean of all measurements.

^††^R_pim_ = ∑_hkl_ [1/(N − 1)] 1/2 ∑_i_ | I_i_(hkl) − [I(hkl)]|**/**∑_hkl_ ∑_i_ I_i_(hkl), where N is the redundancy for the hkl reflection.

^†††^R_work_**/**R_free_ = ∑_hkl_ | Fo − Fc |**/**∑_hkl_ | Fo |, where Fc is the calculated and Fo is the observed structure factor amplitude of reflection hkl for the working**/**free (5%) set, respectively.

In all the ternary complexes, fructose is occupying subsite ‒1 through a net of polar interactions that keep the sugar in a very fixed position at the catalytic pocket, as previously reported^30^. All the polyphenolic compounds are bound by stacking their aromatic rings against Trp105, with additional hydrogen bonds from their OH groups to several residues at the active site directly or through several well-ordered water molecules that are linked to the protein. Moreover, the analyzed compounds present a hydroxyl group linked by a hydrogen bond to the fructose O2, locating such OH group at 3.6–3.8 Å from the fructose C2, a distance that initially is proper for a productive nucleophilic attack leading to transfructosylation. On the other hand, conformational changes are not observed in the residues at the active site upon binding of the different compounds, with the only exception of a switch in the Glu334 side-chain found in the crystal containing EGGC, possibly due to the steric hindrance associated to this bulky compound.

The specific interaction of each inhibitor is depicted in Fig. 4. In the case of *p*-nitrophenol (Fig. 4A), the phenolic hydroxyl linked to the Fru O2 is also linked to Asn79 (ND2), next to the catalytic Asp80 position, and to several water molecules forming a network connected to the protein. This compound is further stabilized in this position through polar interactions of both NO_2_ oxygens to the Gly660 (O) carbonyl, located at the C-terminus of the polypeptide chain. With respect to the crystals containing catechol (Fig. 4B), the OH linked to the Fru O2 is interacting with the ethylenglycol trapped in the crystals, which in turn is linked to the catalytic Glu303 and to Glu334, through a net of water molecules. The other OH of catechol is linked to a water molecule at the same plane that the aromatic ring, increasing the stacking interface to Trp105. Finally, EGGC (Fig. 4C) is accommodated in the catalytic pocket through many atomic interactions. First, the epigallocatechin moiety of EGCG is stacked to Trp105, with the OH next to the Fru O2 being linked to Glu334 carboxylates through water molecules, whilst its second hydroxyl is linked to Gln341(OE1) through a water molecule. The triphenyl ring of epigallocatechin is polar linked to the carbonyl of Leu661 at the C-terminus of the chain. On the other hand, the gallate ring is mainly accommodated by the loop Glu334-Asn342 through direct hydrogen bonds to Gln335 (NE2) and Gln341 (OE1).

The comparison between the three inhibitors binding mode is represented in Fig. 4D and 4E. As can be seen, the positions of catechol and the EGCG diphenolic part of benzopirane are very similar, while the aromatic ring of *p*-nitrophenol is slightly shifted towards the diethylenglycol trapped in the catechol containing crystals. This points to the stacking interaction to Trp105 being the driving force that rules the binding of the different polyphenols, each position being further adjusted by optimizing polar interactions of their hydroxyls substituents with the protein. Furthermore, the structural superimposition of these complexes with the reported complex of pXd-INV-D80A with sucrose (Fig. 4E) can explain the molecular basis of the partial inhibition observed in sucrose hydrolysis by catechol and EGCG, whose hydrolysis rate is 50–65% referred to the control (Table 1). This fact can be compared with the remarkable decrease of this activity produced by *p*-nitrophenol, and the subsequent disappearance of transglycosylation. An inspection to Fig. 4E reveals that the position of *p*-nitrophenol seems to compete directly with the glucose moiety located at +1 subsite of the bound sucrose donor substrate, therefore hindering hydrolysis. In contrast, catechol/EGCG binding position is shifted from subsite +1 therefore tolerating binding of sucrose donor to a higher extent. Interestingly, the two positions observed for the *p*-nitrophenol and catechol/EGCG inhibitors are similar to the two alternative binding modes of the acceptor hydroxytyrosol (HT) previously reported^32^, as it is shown in Fig. 4F. This feature indicates that the binding position of each molecule is not determining its acceptor/inhibitor activity on pXd-INV but, rather, other aspects need to be considered to explain their behaviour, as mentioned below.

The binding of the acceptor (HQ) in the ternary complex is shown in Fig. 5A. As occurs in the other ternary complexes, a hydroxyl oxygen is linked to the Fru O2 and also to the trapped molecule of ethylenglycol, linked further to Glu303 and Glu304 carboxylates through water molecules. The other hydroxyl is connected to Gln341 (OE1) through water molecules. However, it is worth noting that the electron density map shows residual density at lower cut-off indicating some mobility of HQ or, possibly, other minor binding modes. To explore this possibility, the crystals were soaked directly into HQ without pre-incubation with fructose, and the results are shown in Fig. 5B. As it is observed, a Tris molecule from the buffer and an ethylenglycol moiety from the cryoprotectant are mimicking fructose binding at the ‒1 subsite, while a second ethylenglycol molecule is bound similarly to that observed in the ternary complex crystals. On the other hand, the HQ molecule is stacking against Trp105, with one of its hydroxyls being linked to the C-terminal Leu661 carbonyl through water molecules.

Figure 5C represents comparatively the HQ position in the two complexes and the catechol position in the corresponding ternary complex described above. As it is observed, HQ and catechol are similarly located in both ternary complexes, while HQ is shifted in the fructose-free HQ-soaked crystals. This may suggest that binding of HQ can be more flexible than catechol binding, what would be consistent with the residual density observed in the ternary complex with HQ above mentioned. However, and considering that catechol and HQ bind very similarly in the two ternary complexes, it is difficult to decipher their different behaviour as inhibitor or acceptor, respectively. In fact, Fig. 2 showed the presence of a possible transfructosylation product (at low concentration) in the reactions with catechol.

As mentioned before, all the ternary complexes of pXd-INV-D80A with fructose and the different phenolic compounds locate one of the phenolic hydroxyls making polar link to the fructose O2 at 3.6–3.8 Å from the C2, which could enable the ulterior nucleophilic attack by the acceptor leading to transfructosylation. A possible explanation is that the higher mobility of HQ allows the second step of the mechanism to proceed, while catechol remains fixed at its position blocking a putative productive motion of the acceptor substrate. Nevertheless, it is known that an equilibrium between the phenolic-quinone forms can exists in HQ, with a putative associated change in the stacking interaction mode to Trp105, which might influence the distinctive behaviour of this compound as compared to the mostly inhibitory activity of catechol.

### Molecular basis of the pXd-INV transfructosylation mechanism

**Figure 6:**
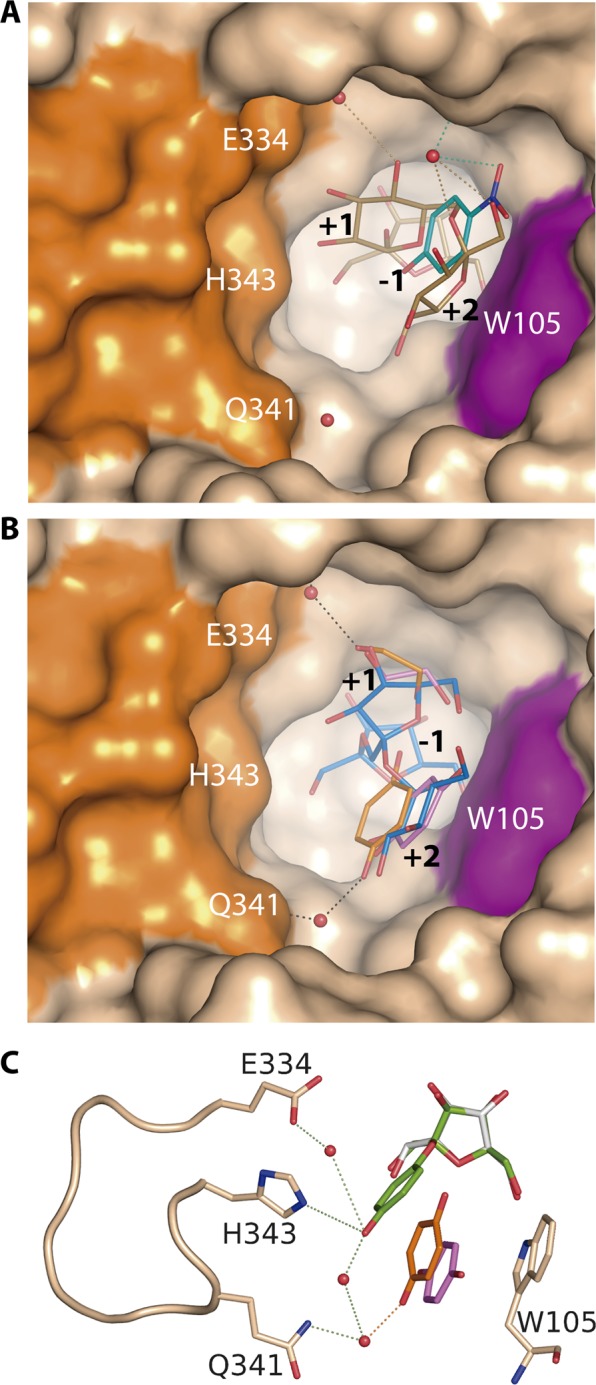
Transfructosylating mechanism of pXd-INV. A detail of the catalytic pocket showing residues relevant in binding: (**A**) *p*-Nitrophenol (cyan) from the ternary complex here reported, superimposed to the neokestose (gold) previously described^30^ (PDB code 5FK7); (**B**) The molecules found in the ternary complex with HQ (orange) and the crystal soaked into HQ (violet), superimposed onto the reported complex with 1-kestose^30^ (marine, PDB code 5FKB). (**C**) A model of the Fructosyl-HQ product (chartreuse) has been manually docked by superimposition of the sugar moiety onto fructose found at ‒1 subsite, the position of the HQ moiety being adjusted by a small torsion of the glycoside bond to satisfy polar interactions with loop Glu334-His343. The molecular surface in A and B is coloured in beige, highlighting Trp105 in magenta and the Glu334-His343 flexible loop in orange. Images created with software Pymol 1.7 (http://www.pymol.org/).

As we have previously described from the analysis of several complexes of pXd-INV-D80A with different β-linked fructooligosaccharides^30^, the subsite +1 is common to all complexes, with Glu334 having a key role in the mechanism, and subsite +2 is mostly created by hydrophobic interactions to Trp105 and polar links to the main chain of the C-terminal segment (through Gly660 and Leu661). However, two different alternative binding modes are observed depending of the substrate and, thus, β(2 → 6)-linked neo-fructooligosaccharides are allocated manly by loop Leu170-Ala172, whereas β(2 → 1)-linked inulin-type fructooligosaccharides are accommodated by loop Glu334-Asn342^30^. In this way, the reported complexes of pXd-INV-D80A with neokestose (gold in Fig. 6A) and 1-kestose (blue in Fig. 6B) revealed the two alternate binding sites of the acceptor sucrose at subsites +1/+2 to generate each trisaccharide series.

According to the complexes here reported, *p*-nitrophenol binds to the *neo-*type subsite with its nitro- substituent interacting with Leu170 through water molecules (Fig. 6A), in a similar way to O2 and O6 from the terminal fructose of neokestose. Considering that neokestose is the main transfructosylating product of pXd-INV, this is the preferred binding site of the acceptor sucrose at the catalytic pocket, which possibly explains the high affinity binding of *p*-nitrophenol to pXd-INV. However, this phenolic compound is fixed at this position by strong polar interaction that may block the progressing of the hydrolytic/transfructosylating enzymatic activity, as explained before. In contrast, catechol, HQ, and EGCG bind at the inulin*-*type subsite +2 (Fig. 6B), occupying the same position that the terminal glucose from 1-kestose. Moreover, one of the hydroxyls of HQ makes a polar link to Gln341 in the ternary complex. Interestingly enough, this binding site is common to both types of compounds, the inhibitors (catechol/EGCG) and the acceptor (HQ). However, the ability of HQ to make polar interactions to Gln341 might explain its different acceptor activity, as Gln341 is included within the highly flexible loop Glu334-Asn342, a region that is disordered in the free enzyme and get ordered upon substrates binding. Therefore, the flexibility of this loop seems crucial for activity and may be directly involved in the production of the fructosylated derivative of HQ.

Thus, an inspection to Fig. 5A shows that the HQ hydroxyl that approximates to the fructose C2 atom is hydrogen linked to the catalytically essential glutamates Glu303 and Glu334 through the two OH from the trapped ethylene glycol molecule. In absence of this diol, two water molecules are playing the same role, as seen in the complex with EGCG (Fig. 4C). Thus, at first, the HQ hydroxyl could be conveniently deprotonated to perform the nucleophilic attack in the second step of the mechanism. Although soaking experiments to get the complex failed, the putative position of the resulting product can be modelled as shown in Fig. 6C, pointing to this secondary product being allocated mainly by the loop Glu334-Asn342. Therefore, this flexible loop may well bring HQ to a productive position to accomplish the nucleophilic attack leading to transfructosylation. However, and in agreement with this binding position being secondary with respect to the preferred *neo*-type, the fructosylated HQ derivative is not produced in high amounts.

On the other hand, and in concordance with the marked inhibition of the sucrose hydrolysis observed in presence of *p*-nitrophenol (Table 1), we hardly observed FOS production along the experiment carried out with this inhibitor (Fig. 2). Furthermore, the significant reduction in hydrolysis rate presented with catechol and EGCG may explain the absence of FOS in the first 30–40 minutes of reaction (Fig. 3), after which these molecules allow moderate FOS production. On the other hand, the dynamic binding of HQ does not impede neither sucrose hydrolysis nor transglycosylation.

## Conclusions

Depending on their chemical nature, phenolics may act as fructosyl acceptors or inhibitors of the β-fructofuranosidase from *X. dendrorhous* (pXd-INV). We measured the effect of such compounds on the hydrolytic and transfructosylating rates, and correlated the results with the crystal structures of the ternary complexes between the inactive mutant pXd-INV-D80A, fructose and the different polyphenols. All the compounds were bound by stacking their aromatic rings against Trp105, with a hydroxyl group linked to the fructose O2 by a hydrogen bond, at an appropriate distance for the nucleophilic attack leading to transfructosylation. The structural superimposition of such complexes with that of pXd-INV-D80A with sucrose helped us explain the partial inhibition observed with several compounds such as catechol and EGCG. We proposed that the acceptor capacity of the different phenolics seems to be determined not only by the binding position of each molecule but by their ability to make flexible polar links with the enzyme. This molecular analysis could be of great value in the design of efficient β-fructofuranosidases that catalyze the synthesis of polyphenol glycosides with bioactive properties, and for the development of inhibitors of related glycosidases implicated in biofuels production or human health.

## Materials and Methods

### Reagents

(-)-Epigallocatequin gallate (EGCG) was acquired from Zhejiang Yixin Pharmaceutical Co. (Zhejiang, China). Glucose, catechol and *p*-nitrophenol were purchased from Sigma-Aldrich (St. Louis, MO, USA). Hydroquinone (HQ) was from Acros Organics (Geel, Belgium). Hydroxytyrosol (HT) was from Seprox (Murcia, Spain). Sucrose was from Panreac (San Fernando de Henares, Spain). Fructose was from Merck (Darmstadt, Germany). 1-Kestose was from TCI Europe (Zwijndrecht, Belgium). Neokestose was synthetized as described in a previous work^26^. All other reagents and solvents were of the highest available purity and used as purchased.

### β-Fructofuranosidase pXd-INV expression and purification

The β-fructofuranosidase from *Xanthophyllomyces dendrorhous* ATCC MYA-131 (Xd–INV) was expressed in *Pichia pastoris* (pXd–INV) as previously reported^39^. Basically, the gene *Xd-INV* (accession no FJ539193.2) fused to the *Saccharomyces cerevisiae* MFα secretion signal sequence was included in plasmid pIB4 (construction QDNS-pIB4) and transformed in *P. pastoris*. The yeast transformant was grown in 50 mL of BMG (1.34% yeast nitrogen base without amino acids, 4 × 10^**−**5^% biotin, 1% glycerol, 50 mM potassium phosphate pH 6.0) during 24 h and protein expression induced in 400 mL of BMM (same as BMG but 0.5% methanol instead of glycerol) for 35 h. The extracellular β-fructofuranosidase activity (about 20 U/mL of culture) was purified using tangential concentration and DEAE-Sephacel chromatography. Active fractions were concentrated using Microcon YM-10 (Amicon) filters (0.7 mL; 4220 U/mL; 5.8 mg/mL) and stored at **−**70 °C. The inactive pXd-INV enzyme (pXd-INV**-**D80A) was obtained using site-directed mutagenesis by substitution of the residue Asp80 acting as nucleophile in the enzyme catalytic mechanism^30^. The inactive protein was expressed in *P. pastoris*, purified and concentrated as above (0.32 mL; 4.2 mg/mL).

### β-Fructofuranosidase activity

The β-fructofuranosidase activity was determined using the 3,5-dinitrosalicylic acid (DNS) assay adapted to a 96-well microplate^46^. Briefly, 45 µL of a 100 mg/mL sucrose solution in 100 mM sodium acetate buffer (pH 5.0) and 5 µL of the enzyme (conveniently diluted) were incubated at 60 °C for 20 min. The quantification of reducing sugars was done with a glucose calibration curve. One unit of activity (U) was the corresponding to the release of one µmol of reducing sugars per minute.

### Transfructosylation assays with pXd-INV

Several phenolic compounds (EGCG, HQ, catechol, HT and *p*-nitrophenol) were tested as acceptors in transglycosylation reactions catalysed by pXd-INV. Transfructosylation reactions were carried out at 60 °C for 2 h. Reaction mixtures contained 0.72 U/mL of pXd-INV, 100 mg/mL of sucrose and 20 mg/mL of the screened phenol. Aliquots were taken out at different times (15, 30, 45, 60, 90 and 120 min), inactivated with two volumes of 400 mM sodium carbonate (pH 11.0) and analyzed by HPLC. A control reaction was performed under the same conditions, but in the absence of the putative acceptor.

### Determination of hydrolysis and transfructosylation rates

The concentration of glucose reflected the total amount of sucrose utilized during the reaction. The rate of hydrolysis was calculated from the amount of fructose released. The rate of transfructosylation was determined subtracting the concentration of fructose from that of glucose, as described in previous works^47–49^. To calculate the initial rates, data was analysed up to approximately 30% of initial sucrose was consumed. The slopes of glucose and fructose formation were determined by the corresponding linear regressions using Sigma Plot 13.0 software. The initial rates of hydrolysis and transfructosylation were calculated by the following equations:

$$\begin{array}{rcl}Hydrolysis\,rate\,(mM\,mi{n}^{-1}) & = & \frac{\Delta [Fructose]}{time}\\ Transfructosylation\,rate\,(mM\,mi{n}^{-1}) & = & \frac{\Delta [Glucose]-\Delta [Fructose]\,}{time}\end{array}$$

### TLC analysis

The screening of phenolic compounds as possible acceptors in transglycosylation reactions with pXd-INV was performed by Thin Layer Chromatography (TLC) on silica gel plates with fluorescent indicator (Polygram SIL G/UV254, Macherey-Nagel, Düren, Germany). A mixture of ethyl acetate and methanol (3:1 v/v) was used as eluent. Phenolic compounds were observed under UV light and sugars were revealed submerging plates in a general staining solution [(NH_4_)_6_Mo_7_O_21_·4H_2_O + Ce(SO_4_)_2_ in 10% H_2_SO_4_], drying, and heating for a few minutes.

### HPLC analysis

High Performance Liquid Chromatography (HPLC) was performed using a quaternary pump (Agilent Technologies model 1100, Santa Clara, CA, USA) coupled to a Waters Spherisorb amino column (250 × 4.6 mm) from Waters (Milford, MA, USA). The column was kept at 30 °C and samples were automatically injected with a Hitachi L-2200 autosampler (Hitachi, Tokyo, Japan). Injection volume was 10 µL. The initial mobile phase was CH_3_CN:H_2_O 82:18 (v/v), which was kept for 6 min. Then, a gradient to CH_3_CN:H_2_O 70:30 (v/v) was performed in 1 min and this composition was maintained for 10 min. The flow rate was 1 mL/min. Phenolic compounds were analyzed with a photodiode array detector (PDA, Varian ProStar, Palo Alto, CA, USA) and sugars were detected by an evaporative light scattering detector (ELSD 2000ES, Alltech, Lexington, KY, USA). ELSD conditions were set at 83.5 °C and a nitrogen flow of 2.2 L/min. Chromatograms were analyzed employing the Varian Star LC workstation 6.41 (Varian, Palo Alto, CA, USA).

### Purification of fructosyl hydroquinone

The fructosylation reaction with hydroquinone was scaled-up to 3 mL. Sucrose concentration, temperature and buffer were the same as described in the transfructosylation assays, except for phenol concentration that was increased to 50 mg/mL. Fructosyl-hydroquinone (Fru-HQ) was purified by semipreparative HPLC using the same equipment employed in analytical chromatography with the addition of a three-way flow splitter (Accurate, LC Packings, Amsterdam, Netherlands). The column employed was a Liquid Purple amino (250 × 10 mm, Analisis Vinicos, Tomelloso, Spain). Injection volume was increased to 50 µL. The initial mobile phase was CH_3_CN:H_2_O 75:25 (v/v), which was kept for 6 min. Then, a gradient to CH_3_CN:H_2_O 70:30 (v/v) was performed in 1 min and this composition was maintained for 6 min. The flow rate was 5 mL/min. Fractions containing the fructosylated derivative were pooled and the solvent was eliminated by rotary evaporation.

### Mass spectrometry

Mass spectrometry (MS) of the transfructosylation product obtained with HQ was assessed using a mass spectrometer with hybrid QTOF (quadrupole time of flying) analyzer (model QSTAR, Pulsar i, AB Sciex, Framingham, MA, USA). The sample was analyzed by direct infusion and ionized by electrospray (with methanol as ionizing phase) both in positive and negative reflector modes.

### Crystallization and X-ray structure determination

As a last step of purification, pXd-INV-D80A mutant was deglycosylated with Endo H (England Biolabs) prior to crystallographic experiments. Then, released sugar and Endo H were eliminated by spinning with a 50 kDa Amicon (Millipore) and the protein was subsequently concentrated to 8 mg/mL with a 10 kDa Amicon, in 20 mM Tris buffer (pH 7.0). Crystals were grown as described previously^30,31^. The complexes were obtained by soaking crystals in precipitant solution (1.3 M sodium citrate) supplemented with 20 mM fructose for 10 min, followed by immersion in precipitant solution plus 40–100 mM of the following compounds: *p*-nitrophenol (4 hours), EGCG (o/n), hydroquinone (o/n), or catechol (5 days). For the other phenols, we also tested direct soakings eluding preliminary incubation with fructose, but only pXd-INV-D80A complexed with hydroquinone was achieved after soaking crystals for 3 days into the precipitant solution supplemented with 100 mM HQ. Finally, the crystals were soaked in mother liquor supplemented with purified 85 mM fructosyl-hydroquinone (see production method above) for a time ranging from few hours to several days. In all cases, a sucrose molecule was found at the active site, possible derived from slight contamination of the fructosylated product.

For data collection, all pXd-INV-D80A crystals were transferred to cryoprotectant solutions consisting of mother liquor plus 10% (v/v) ethylene glycol before being cooled to 100 K in liquid nitrogen, and diffraction data were collected using synchrotron radiation on the XALOC beamline at ALBA (Cerdanyola del Vallès, Spain). Diffraction images were processed with XDS^50^ and merged using AIMLESS^51^ from the CCP4 package^52^. All pXd-INV-D80A complexes were indexed in the P2_1_2_1_2 space group with two molecules in the asymmetric unit and 70% solvent content within the unit cell. The data-collection statistics are given in Table 2. The structures of the pXd-INV-D80A complexes were solved by difference Fourier synthesis using the coordinates of the native protein (PDB code 5ANN). Crystallographic refinement was performed using the program REFMAC^53^ within the CCP4 suite with flat bulk-solvent correction, maximum likelihood target features and local non-crystallographic symmetry (NCS). Free R-factor was calculated using a subset of 5% randomly selected structure-factor amplitudes that were excluded from automated refinement. At the later stages, ligands were manually built into the electron density maps with COOT^54^ and water molecules were included in the model, which ‒combined with more rounds of restrained refinement‒ yielded the R factors listed in Table 2. The figures were generated with PyMOL^55^. Coordinates for all the structures have been deposited in the Protein Data Bank under accession numbers 6FJE, 6S2H,6S2G, 6S3Z and 6S82 (Table 2).

## Supplementary information

Supplementary information
