# Supplementary material for: Deciphering the molecular specificity of phenolic compounds as inhibitors or glycosyl acceptors of β-fructofuranosidase from Xanthophyllomyces dendrorhous
Source: Sci Rep. 2019 Nov 25;9:17441. doi: 10.1038/s41598-019-53948-y (PMC6877581; doi:10.1038/s41598-019-53948-y)
Supplement: Supplementary file 1 — Supplementary information [file 41598_2019_53948_MOESM1_ESM.pdf]

Deciphering the molecular specificity of phenolic compounds as inhibitors or glycosyl acceptors of β-fructofuranosidase from *Xanthophyllomyces dendrorhous*

M. Ramírez-Escudero[a], N. Míguez[b], M. Gimeno-Pérez[c], A.O. Ballesteros[b], M. Fernández-Lobato[c], F. J. Plou[b,*] and J. Sanz-Aparicio[a,*]

[a] *Macromolecular Crystallography and Structural Biology Department, Institute of Physical-Chemistry Rocasolano (CSIC), Serrano 119, 28006 Madrid, Spain*

[b] *Institute of Catalysis and Petrochemistry (CSIC), Marie Curie 2, 28049 Madrid, Spain*

[c] *Centre of Molecular Biology Severo Ochoa (CSIC-UAM), Autonomous University of Madrid, 28049 Madrid, Spain*

Figure S1. Mass spectrometry ESI-QTOF analysis of HQ fructoside (4-hydroxyphenyl-β-D-fructofuranoside).

Fig. S1

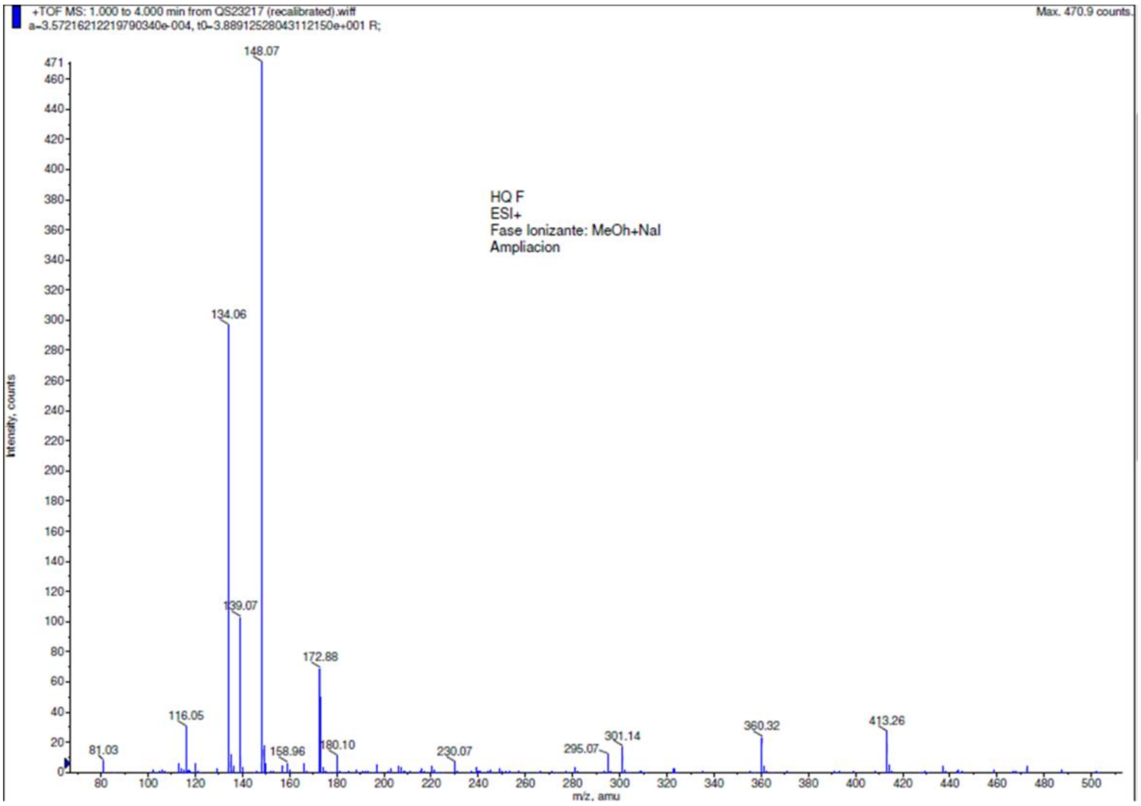
+TOF MS: 1.000 to 4.000 min from QS23217 (recalibrated).wiff
a=3.57216212219790340e-004, t0=3.88912528043112150e+001 R;
Max. 470.9 counts.
HQ F
ESI+
Fase Ionizante: MeOh+NaI
Ampliacion
148.07
134.06
139.07
172.88
116.05
81.03
158.96
180.10
230.07
295.07
301.14
360.32
413.26
Intensity, counts
m/z, amu
